# Prebiotic Synthesis of ATP: A Terrestrial Volcanism-Dependent Pathway

**DOI:** 10.3390/life13030731

**Published:** 2023-03-08

**Authors:** Xin-Yi Chu, Hong-Yu Zhang

**Affiliations:** 1 Hubei Key Laboratory of Agricultural Bioinformatics, College of Informatics, Huazhong Agricultural University, Wuhan 430070, China; 2 Qian Xuesen Collaborative Research Center of Astrochemistry and Space Life Sciences, Institute of Drug Discovery Technology, Ningbo University, Ningbo 315211, China; 3 Development Research Center of Ordos, Ordos 017000, China

**Keywords:** adenosine triphosphate (ATP), origin of life, volcanism, RNA world, protein cofactor

## Abstract

Adenosine triphosphate (ATP) is a multifunctional small molecule, necessary for all modern Earth life, which must be a component of the last universal common ancestor (LUCA). However, the relatively complex structure of ATP causes doubts about its accessibility on prebiotic Earth. In this paper, based on previous studies on the synthesis of ATP components, a plausible prebiotic pathway yielding this key molecule is constructed, which relies on terrestrial volcanism to provide the required materials and suitable conditions.

## 1. Introduction

Composed of adenine, ribose, and a triphosphate group, adenosine triphosphate (ATP) is among the most important molecules to Earth life. It is the universal “energy currency”, one of the units of RNA, the activator of amino acids in protein synthesis, and the most prevalent protein cofactor [1,2]. ATP is also an important regulator, through working as the substrate of regulatory phosphorylation and controlling biomacromolecule aggregation as a hydrotrope [3,4]. Bearing these fundamental functions, ATP should have existed in the last universal common ancestor (LUCA).

However, due to the relatively complex structure and the demand for relatively rare material (phosphorus, in particular), the prebiotic synthesis of ATP is a multi-step process that needs to overcome various obstacles. Therefore, the availability of ATP on primitive Earth is questionable, and some theories suggested that ATP may not be the first player of its current roles during the origin of life. Thioester has been suggested to work as an energy reservoir and supported the expansion of primitive metabolic networks [5,6]. Abundant metal compounds such as FeS can be used as coenzymes for early proteins [7]. Inorganic phosphates were suggested to be the prebiotic phosphorylating agents [8]. ATP may not even be the unit of the earliest informational polymer; nucleic acid analogs such as depsipeptide nucleic acids are attractive challengers to the RNA-first scenario [9].

As a necessary piece of the puzzle, the role of ATP played in the origin of life is fundamental and intriguing. Although the de novo synthesis of ATP has not been reported, the origin of its components, including ribose, adenine, and the triphosphate group has been extensively studied. In this paper, based on previous knowledge, we investigate what environments on primitive Earth may enable the steps in ATP synthesis and try to construct a pathway by which they can collaborate to produce ATP. Based on this premise, we briefly discuss how ATP is involved in the origin of life.

## 2. Source of Materials Required for ATP Synthesis

Like most other biological molecules, ATP is composed elementally of hydrogen (H), carbon (C), nitrogen (N), oxygen (O), and phosphorus (P). The first four elements are abundant in the solar system (5.13 × 10^6^, 7.60 × 10^5^, 5.53 × 10^4^, and 7.53 × 10^6^ atoms per 10^6^ atoms of Si), while P is relatively less (8.34 × 10^3^ atoms per 10^6^ atoms of Si) but still not rare [10]. In addition to being in sufficient amounts, these elements also need to exist in proper forms and environments for biomolecular synthesis to take place. For example, inert diamonds, graphite, and carbonaceous minerals are not good carbon substrates for prebiotic reactions. Fortunately, there are multiple sources of hydrogen, carbon, oxygen, and nitrogen that exit in volatile forms, which are more likely to participate in biomolecular synthesis reactions. For instance, N_2_, CO_2_, and H_2_O are major components of inferred primitive atmospheres of rocky planets like Earth, Mars, and Venus [11]. H_2_O also makes up the ocean of Earth, primitive Mars [12], and Jupiter’s satellite, Europa [13]. NH_3_ exists in the atmosphere of Jupiter and Saturn [14], their satellites like Titan [15], the ocean (companioning with H_2_O) of Uranus and Neptune [16], and Pluto’s surface [17]. Simple organics like CH_4_ are also common in the solar system. Massive quantities of CH_4_ form oceans on ice giant planets and Titan [16]. As will be shown, these substances are capable of producing important molecules such as aldehydes and cyanides, which could be the foundation of ribose and nucleobase synthesis.

**Figure 1:**
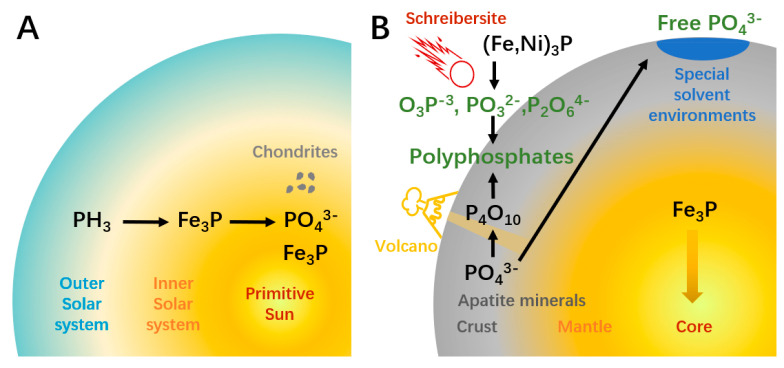
The source and potential solution of the phosphorus problem. (**A**) In the nebula that formed the solar system, phosphorus mainly exists in the form of PH_3_. The heavy element Fe was enriched in the inner solar system which reacted with PH3 to form Fe_3_P. Part of Fe_3_P was oxidized to phosphate minerals (PO_4_^3−^) during the formation of celestial bodies like chondrite. (**B**) On the newly formed earth, 80% of the phosphorus combined with Fe and sunk to the core. Crust phosphorus mainly exists in the form of insoluble apatite minerals, which is difficult for participating in prebiotic reactions. Lakes or pools of formamide, carboxylic acids, or carbonate solutions can extract PO_4_^3−^ from minerals. Volcanic activity can generate polyphosphates, which is a class of active phosphorylation reagents. Schreibersites that fall to Earth can react with water and generate hypophosphite, phosphite, and hypophosphate, which are active phosphorylation reagents and can be further oxidized to produce polyphosphates.

Different from the other four elements, reactive phosphorus is less available for the prebiotic synthesis of organophosphorus compounds like ATP. This obstacle to the origin of life is the so-termed “phosphorus problem”. The root of the problem can be traced to the formation of solar galaxies (Figure 1A). In the nebula stage of the solar system, phosphorus mainly existed as PH_3_ gas. The siderophile property induces PH_3_ tending to react with iron and generated the earliest phosphorus mineral schreibersite ((Fe,Ni)_3_P), which removed almost gaseous phosphorus in the inner solar system in around 10,000 years [18]. In the modern solar system, abiotic PH_3_ can only be found in gas giant planets. Therefore, unlike other biogenic elements that mainly exist in volatile forms, phosphorus must be “extracted” from minerals before participating in the synthesis of biomolecules. During the system evolution, mineral particles accumulate into larger objects like chondrites [19]. The thermal metamorphism and equilibration of chondrite components change the redox state, oxidizing part of reduced phosphorus to phosphate minerals. On planetary celestial bodies such as Earth, the complex geological process caused further differentiation and uneven distribution of phosphorus (Figure 1B). The siderophile property induced a large amount of phosphorus to sink to Earth’s core with iron. It is estimated that the core contains 80% of the total phosphorus on Earth. In the core and the metal-saturated deep mantle (below 250 km), phosphorus mainly exists as P^0^ or P^1−^, while phosphorus left in the upper silicate mantle is oxidized (P^5+^). Near the surface of the earth, phosphorus manifests as an incompatible element during the repetitive mantle melting. As a result, most crust phosphorus exists in the apatite mineral group (Ca_5_(PO_4_)_3_OH) [19]. The solubility of this mineral is low (~10^−6^ M), which means that the phosphate ions (H_2_PO_4_^−^ or HPO_4_^2−^, depending on pH) are hard to extract from minerals. Moreover, in organic phosphorylation products, phosphorus exists in the form of R-OPO_3_H^−^, which means that metaphosphate (PO_3_^−^) is the reactive species in phosphorylation. To form metaphosphate from phosphate, the removal of H_2_O or OH^−^, a dehydration process is needed, which is incompatible with the aqueous solutions required for most prebiotic reactions [20].

Solutions to the phosphorus problem can be classified into two categories. The first possible solution is enhancing the extraction of phosphorus from minerals, which depends on special solvent environments (Figure 1B). Changing the solvent is a common way to enhance solubility in modern chemistry. Formamide can release phosphate from various minerals [21]. The eutectic solvent consisting of choline chloride and urea enables the high yields of phosphorylated nucleosides [22]. However, it is unlikely that these substances ever reached sufficient concentrations and purity on Earth to be used as prevalent solvents [20]. Other studies used chelation and condensing agents to promote the dissolution and reaction of inert minerals. Carboxylic acids can elevate mineral solubility by chelate cations [23]. Despite the successful examples, for the organic chelating agent to be effective, its concentration is presumed to need to be 10^−2^ M, which may have been difficult to achieve on primitive Earth [20]. A more recent version of this solution uses carbonate, a possibly more abundant substance on prebiotic Earth, as the condensing agent, which suggests a high concentration of phosphate can be found in carbonate-rich lakes [24].

The second solution to the phosphorus problem is finding possible reactive phosphorus (Figure 1B). Polyphosphates are ideal phosphorylation reagents. In addition to higher solubility, unlike reactions using phosphates, phosphorylation using polyphosphates does not require the removal of water or hydroxyl groups, which means it can occur in aquatic conditions [20]. Trimetaphosphate could be the most studied polyphosphate, which can phosphorylate sugars, glyceric acid, amino acids, and nucleosides under moderate conditions [25,26]. A weakness of the polyphosphate-based pathway is that the corresponding minerals are rare on Earth. Volcanic fumarole may have been a major source of polyphosphate on primitive Earth. Yamagata et al. showed that a high temperature of over 1000 °C can transform phosphate minerals to P_4_O_10_; the latter can release trimetaphosphate and other polyphosphates when reacting with water [27]. The half-life of polyphosphates ranges from days to years [28,29], implying a need for constant but mild volcanic activity to enable a stable supply without damaging surrounding organic matters. A “living” example of this environment is the active volcano Mount Usu, active since 1663 [30], where polyphosphate has been detected in the gas released [27]. This kind of condition appears in different geological eras and may be more prevalent on the young Hadean-Archaean Earth.

Another attractive option is phosphide, which may preserve in meteorite mineral schreibersite [(Fe,Ni)_3_P]. This reduced phosphorus mineral reacts with water and produces phosphorous compounds of varying degrees of oxidation, including soluble hypophosphite, phosphite, and hypophosphate [31]. Further oxidation can also produce polyphosphates such as hypophosphate and triphosphate. Phosphite (PO_3_^2−^) is a major product of the reaction between schreibersite or Fe_3_P and water. As a strong electrophile, PO_3_^2−^ can react with the OH group of the organics to phosphorylate the latter [31]. This reaction has been verified in the phosphorylation of glycerol and nucleosides in aqueous solution [31,32] and liquid sulfur dioxide [33], suggesting the potential role of extraterrestrial phosphorus in the origin of life. Phosphite is stable in the reducing environment of primitive Earth, with a half-life between 10^8^ and 10^10^ years [34]. A study of the composition of rocks from different ages found that phosphite is only present in the oldest 3.52 Ga-years-old early Archean samples, which may be rooted in the enstatite chondrites during the bombardment that occurred about 4.5–3.8 Ga [35]. The disappearance of phosphite in samples younger than 3.4 Ga implies a time window for its availability.

The two solutions to the phosphorus problem imply that the element available to the origin of life on Earth may depend on volcanic activity or random meteorite impacts rather than conventional atmospheric or aquatic components. In the context of substance supply, phosphorus seems to be the bottleneck of the pathway to ATP. As seen above, the substance needed for ATP synthesis may be available on primitive Earth. In light of these potential substance sources, the next section will discuss how they might support plausible pathways to ATP.

## 3. Plausible Pathways to ATP

Composed of ribose, nucleobase, and phosphate group, the structure of nucleotides determines that their synthesis must go through multiple steps, whether in biotic or abiotic style. The related reactions have been summarized in recent excellent reviews [36,37,38]. We briefly introduce the representative reactions and discuss how might they cascade together to eventually lead to ATP. Although not necessarily a precondition, the synthesis of the parts of ATP will be discussed first.

### 3.1. Adenine Synthesis

Adenine was the first nucleobase to be synthesized abiotically, which was obtained in 1960 through heating ammonium cyanide to below 100 °C [39]. Despite the reactant (up to 11 M) and the low yields (about 0.5%), the pioneering work confirmed that adenine can be obtained from simple small molecules. Later studies revealed the key polymerization processes of HCN in this kind of reaction [37]. Although the generation of HCN may have been common on primitive Earth, which can be achieved by the electrical discharges in the N_2_-CO_2_ atmosphere or supplied by the meteorite impacts, the high concentration (over 1 M) demanded by these early studies is difficult to achieve due to its instability and high reactivity [40]. Therefore, the preservation and concentration of HCN are necessary conditions for nucleobase synthesis using it as a starting material.

One potential way to stabilize HCN is to store it in less reactive salts. Recently, Sasselov et al. proposed a plausible scheme for the prebiotic cyanide chemistry: HCN generated in the atmosphere washed into mineral-rich shallow waters, at depths beyond the reach of UV, HCN reacts with Fe^2+^ to form ferrocyanide and exists stably [41]. When water was evaporated, ferrocyanide formed mixed salts with Ca and Mg, and precipitated, which could be seen as a concentration step. These accumulated cyanide salts can be converted to CaCN_2_, KCN, NaCH, and Mg_3_N_2_ under intense heat like volcanic activity and large impacts. When encountering near neutral solutions, they generate HCN, NH_3_, and H_2_CN_2_. Under mid-range UV irradiation, the reactions among these feedstocks and other prebiotic compounds like SO_2_ could be an efficient way to generate the precursor of nucleobases, amino acids, and lipids.

Compared to HCN, HCN-polymer is more stable and has diverse hydrolysis patterns under different conditions, and thus is considered to be a plausible source of many biotic molecules. The formation of HCN-polymer does not necessarily depend on the high concentration of HCN. At a temperature below −20 °C, an HCN tetramer (DAMN) was generated from a dilute solution (0.1 to 0.001 M). A subsequent room temperature melting step produced adenine, albeit in very low yields [42]. Based on this finding, the Miller group carried out a series of long-term experiments, in which 0.1 M NH_4_CN or a mixture of HCN and NH_3_ were frozen from 2 months to 27 years [43,44,45]. Cyanide polymer, adenine, guanine, and other compounds were found in the melt materials. Heating the reaction mixture with acid (6 N HCl) or alkali (0.01 M phosphate, pH = 8) solution can significantly enhance the production of adenine to 2.9 and 1.2%, though the acid hydrolysis also degenerates half of the generated adenine [45]. These studies showed a “cold” origin of nucleobases, in which the products can be stored for longer periods and accumulated in larger quantities to sustain subsequent reactions. Interestingly, HCN-polymers can also form under an alkaline hydrothermal environment. Heat 0.15 M HCN over 100 °C, pH = 10 generated stable polymers [46,47]. Theoretically, under such an environment, HCN polymers can be used to synthesize nucleobases, and further experimental research is required [48].

Formamide which is generated during HCN hydrolysis was intriguing because of its potential to reach high concentration and boiling point, which means it can suffer high-temperature reactions. Heating formamide between 140 and 200 °C gave high yields of purines [37,49]. Several minerals can change the composition and yield of the products: adding CaCO_3_, TiO_2_, phosphate minerals, and iron sulfide minerals during heating facilitate the production of adenine [50,51]. Ultraviolet light also has such an effect [52]. A disadvantage of formamide is its high hygroscopicity, which means that formamide mainly exists in the mixture with water. Nucleobases cannot be effectively produced in formamide solutions with more than 10% water content [51]. Some special conditions can remove water from formamide. At a temperature between the freezing point of formamide (2.25 °C) and water, formamide froze and sank to the bottom of the water body. When the temperature continues to drop below 0 °C, the top water layer also froze, which may be removed by a following sublimation or ablation process [53]. Purification of formamide could also be achieved in a fractional distillation-like manner with a suitable temperature gradient from 10 to 70 °C [54].

Beyond HCN-related materials, adenine can also be obtained from the combinations of N_2_, ammonia, methane, CO, and water, which demand high energy input such as electron beam, electric discharge, laser-driven plasma, or high-temperature plasma [37]. In some of these processes, HCN is also generated and worked as an intermediate product [55]. Adenine and other nucleobases can also be derived from more complex starting materials such as urea and amino acid derivatives, as summarized by Yadav et al. [37]. These pathways should still essentially be traced back to the aforementioned simple small molecules and are not discussed in this paper.

Among the five nucleobases used in current life, adenine seems to be the most readily abiotically synthesized through the HCN-based pathways, which may be the reason for its more widespread use in life. Adenine has a half-life of 1 year at 100 °C in solution and can be extended to decades at lower temperatures [56]. If out of the water environment, the half-life of dry adenine can be over 10^6^ years, enabling geological time scale accumulation [57].

### 3.2. Ribose Synthesis

The formose reaction discovered in 1861 has long been of interest as a plausible prebiotic sugar synthesis pathway [58]. In the canonical formose reaction (in an alkaline solution environment, catalyzed by the minerals), formaldehyde or other low-carbon aldol polymerize and generates a complex mixture of mono- and polysaccharides. The materials and conditions required for the reaction to occur are plausible: formaldehyde can be generated in the photochemical and electrochemical reactions from CO_2_ in the primitive atmosphere, or delivered by meteorites or comets [59]. The alkaline solution environment can be provided by hydrothermal vents. However, using formose reaction as the ribose source to synthesize ATP needs to solve several problems [37]. First, while the reaction demands a relatively high concentration of formaldehyde (about 0.1 M), it is easily converted to formate and methanol in solution. Second, the kinetics and products of the reaction are complex. The composition of the reaction product is greatly affected by the reaction time, excessive reaction time reduces the yield of sugars; and in any case, ribose is always a small part of the product. Third, the aimed product, ribose, is also unstable, with a half-life of less than one hour at 100 °C. Therefore, for the accumulation of ribose based on formose reaction, an environment with an adequate supply of formaldehyde as well as the ability to enhance ribose production, selection/purification, and preservation would be ideal.

Among the possible sources of formaldehyde on primitive Earth, the photochemical reaction in the atmosphere may be the most prevalent one. Under the action of hydrogen and light, CO_2_ was reduced to methane in several steps. According to a pioneer model, formaldehyde produced in the atmosphere was rained into the ocean and accumulates to 10^−3^ M over millions of years [60]. Volcanic activity can facilitate this process, which provides large amounts of CO_2_ and hydrogen, and simultaneously induce electrics—the energy source of electrochemical synthesis of formaldehyde. Formaldehyde from atmospheric sources should be more abundant in shallow waters, such as terrestrial hot spring systems and intertidal zone near volcanoes. In the opposite direction, deep-sea hydrothermal vents are “hot” areas in the origin of life studies because of their ability to produce abundant organic matter. Theoretical and simulation studies suggested that hydrothermal vents can generate formaldehyde through methanediol reduction and dehydration [61], or catalyze formose reaction in CaCO_3_-based chemical gardens [62], though there is no conclusive evidence that these processes occur near modern hydrothermal vents.

After ensuring the source of raw materials, the next issue is enhancing ribose production. Several minerals could be helpful. Borate and silicate minerals can stabilize sugars by binding them with released ions, which not only stabilize the sugar but may also modulate the reaction process [63,64]. However, the availability of borate ions in primitive Earth is questionable, due to the scarcity of the element in the earth’s crust. The selectivity of silicate on ribose is controversial. Moreover, these processes may result in complex that cannot undergo further reactions or so-called “dead-end” complex [37]. The participation of phosphors also helps to enhance ribose formation. Hydroxyapatite was reported to enhance ribose production, but it is still not a major product [65]. Using phosphorylated aldehydes as starting materials, products that are less complex than the conventional formose reaction can be obtained. Mixing glycolaldehyde-2-phosphate with formaldehyde in 2 M NaOH, up to 15% ribose 2,4-diphosphate can be generated at room temperature [66].

Purifying or selecting ribose from complex products could be another solution. Recently, Zhao and Wang’s experiments showed that metal-doped-clays (MDC), which can be widely speared on the primitive Earth, is a new solution [67]. They found that clays including kaolin, montmorillonite, and mica which doped Cu^2+^, Ca^2+^, or Fe^2+^ have a significant preference for the retention of ribose in the formose reaction products. This effect can separate ribose from the alkaline reaction solution thereby enriching and protecting it to a certain extent. Silica mineral surface is also able to absorb and protect ribose and prefers the furanose form [68].

Beyond the formose reaction, the “glyoxylate scenario” is another possible prebiotic pathway to ribose. Instead of formaldehyde, the reaction started with glyoxylate and its dimmer dihydroxyfumarate (DHF) and generate ketoses and sugar acids in a more “clean” way. The experiment showed that the glyoxylate scenario is feasible under lithium or cesium catalysis, at pH ≈ 8–9, 4 °C [69]. Whether this more specific approach to producing ribose precursors can be implemented on primitive Earth requires more investigation. Glyoxylate may be easily generated by the reductive conversion of CO_2_, but the prebiotic synthesis of DHF is only theoretical. Moreover, the catalysts currently used are not abundant on Earth, more common materials will provide a higher probability that the scenario occurred on primitive Earth.

No matter how ribose was generated on the primitive Earth, the short half-life in minutes in aquatic environments is an obstacle to its participation in the synthesis of biomolecules [70]. The alkaline and normally hot condition in formose reaction is especially harmful to ribose. Adsorption of ribose to the solid phase can extend its half-life to hours, a limited effect [68]. Low temperature can preserve ribose solution for decades [70], but it does not seem to be compatible with volcanic environments. It may be a more plausible scenario that ribose is used to synthesize nucleosides as soon as possible after production.

### 3.3. Adenosine Synthesis and Phosphorylation

Early studies tried the synthesis of nucleosides by heating the mixture of ribose and nucleobases. This simple method works in adenosine under the catalysis of MgCl_2_ and trimetaphosphate, achieving a yield of about 4% [71]. Phosphorylated ribose is the substrate of the biological synthesis of nucleosides and performed better in abiotic reactions. The attempts to synthesize nucleosides with phosphorylated ribose have been partly successful in adenosine. A 15% yield of adenosine-2′-phosphate was obtained by heating the dilute solution of ribose-1,2-cyclic phosphate and adenine to dryness at 85 °C in the presence of calcium chloride [72]. However, this kind of method cannot produce other nucleosides efficiently [73]. Several environmental factors, such as silica surface, kaolinite, and aqueous microdroplet, were reported to facilitate the condensation of the parts of nucleosides and nucleotides, but inconsistent results of the constitution or structure of the products from different methods appeared in some studies, suggested that they should be regarded with caution [37]. Moreover, this kind of method also cannot produce pyrimidine nucleosides.

Beyond condensing ribose (or phosphorylated ribose) and nucleobase, there are alternative methods to produce nucleosides. By constructing a cytidine bit-by-bit on a phosphorylated ribose with cyanamide, Sanchez and Orgel achieved the synthesis of pyrimidine nucleosides firstly, though the yield of the target product β-cytosine was 5% [74]. The key intermediate in the reaction, amino-oxazoline, inspired other studies, represented by the Sutherland groups. In their system, amino-oxazoline originates from HCN and H_2_O, and then produces pyrimidine nucleosides [75]. This pathway is also a part of the HCN-based reaction network, which produces multiple amino acids and lipid precursors [76]. In an altered route, instead of amino-oxazoline, 2-thiooxazole was synthesized first by glycoaldehyde and thiocyanic under heating and dehydration at 80 °C [77]. Interestedly, this sulfur-dependent scenario can generate both purine and pyrimidine nucleotides, through experiencing acidic or basic conditions. However, the final products are generated by heating with phosphate in a urea/formamide mixture, this condition may not be prevalent on primitive Earth.

If it is assumed that the phosphorus problem can be solved on the primitive Earth as mentioned above, the phosphorylation of nucleobase should be available. Several different phosphorylation reagents have been tested and summarized by Kitadai and Maruyama [36]. Basically, adenosine monophosphate (AMP) can be generated from adenosine by heating with phosphate or polyphosphates in dry or aquatic conditions. Heating the adenosine solution containing urea, MgSO_4_, K_2_CO_3_, or NH_4_OH; and the analogs of schreibersite (Fe_3_P and Fe_2_NiP) also generate AMP [32]. The earliest abiotic synthesis of ATP in the context of the origin of life was performed in 1963, in which ethyl metaphosphate and adenosine dilute solution (less than 1 mM) was treated with UV irradiation at 40 °C, which generated 0.5% ATP, 0.2% ADP, and 0.1% ATP in one hour [78]. The subsequent studies showed that trimetaphosphate is arguably the most successful phosphorylation reagent for ATP synthesis. Kept the solution containing 0.05 M adenosine and trimetaphosphate at room temperature and pH 8-12 for ten days can produce 91.8% AMP and 3% ATP. High pH (12-14) promotes the production of ATP (9.9%) but degrade AMP (8.5%) [79]. Dehydration and metal ions promote the phosphorylation reaction. Under the catalyzing of Ni ions and four rounds of wet-dry cycles at 37 °C in two weeks, the mixture of 0.02 M adenosine and 0.2 M sodium trimetaphosphate generates different adenosine phosphates including 13% ATP [80]. The reaction catalyzed by Ag ions reached a 23.7% ATP yield, though this rare element cannot participate in prebiotic reactions on a large scale. ATP can also be generated in a single aqueous phase or solid phase reactions, albeit less efficiently. Certainly, ATP can be generated from AMP and ADP, with similar conditions in AMP formation [36]. The difficulties and solutions in ATP synthesis are summarized in Table 1.

### 3.4. A Terrestrial Volcanism-Dependent Pathway to ATP

As so far, we summarized the possible steps in ATP abiotic synthesis and discussed the environments that may support them. Terrestrial volcanism could be an important contributor to linking these steps together to construct a pathway connecting the basic components of primitive Earth and ATP. During volcano activity, the generated large amounts of hydrocarbons, CO, and CO_2_ altered the local atmospheric composition. Combined with triggered lightning, the atmospheric environment around active volcanoes should be suitable for HCN and formaldehyde production, which serve as the source substances of nucleobase and ribose. Polyphosphates, especially trimetaphosphate from milder volcanic activity enable phosphorylation reactions. The geothermal effect can promote reactions that require heating.

**Figure 2:**
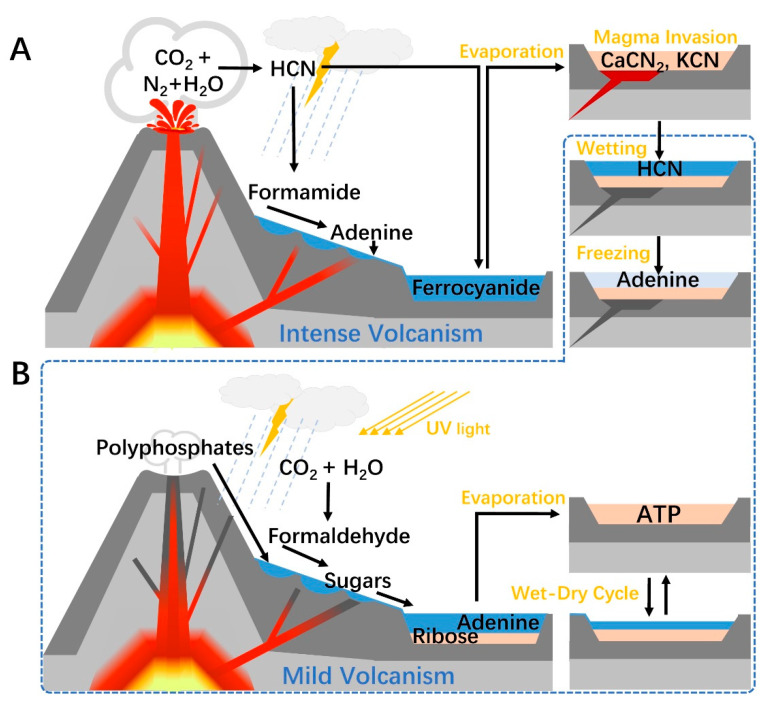
A terrestrial volcanism-dependent pathway for prebiotic ATP synthesis. (**A**) Adenine synthesis. During intense volcanism, HCN is generated in the atmosphere around an active volcano and then partially hydrolyzed to formamide. As the mixture of formamide and water washed down the slope, the increasing temperature gradient, created by geothermal activity, removed most of the water by fractionation. Adenine is produced in the hot formamide pond and is washed into cooler water. Alternatively, after HCN enters a mineral-rich pool, it forms ferrocyanide with ferrous ions and is converted into soluble cyanide salts after suffering the intense heat caused by magma invasion. During mild volcanism, cyanide salts revert to HCN when encountering water, and the latter polymerizes under a freezing condition to give adenine. (**B**) Ribose and ATP synthesis. Formaldehyde is produced in the atmosphere by photochemical and electrochemical reactions, then washed into the ponds around the volcano along with the polyphosphates from the fumarole. Formose reaction occurred in these pounds, then the generated ribose may be separated from the sugars by minerals along the way downstream. Finally, the substrates are assembled in a pool or lake at the foot of the volcano, and ATP can be synthesized during wet-dry cycles.

Centering on volcanic activity, a plausible pathway for prebiotic ATP synthesis could be constructed. In the first stage, the volcano is highly active (Figure 2A), large amounts of HCN and formamide were produced in the surrounding atmosphere and washed into lakes or lagoons by rain and rivers. A suitable temperature gradient may form on the slope of the volcano, where the water was gradually evaporated to obtain concentrated formamide. By mineral catalysis, the remained formamide can yield adenine which can be preserved in the dry basin. This process may also trigger the HCN preservation scenario supposed by Sasselov et al. [41], if magma invades the basin, though it can also degrade the already formed adenine. In the second stage, the volcanic activity turns mild (Figure 2B). Cyanide-rich basin generated in the previous stage was again filled with water and released HCN. Freezing of water bodies in winter can induce the polymerization of HCN and produce adenine. A terrestrial hydrothermal environment may also appear near the volcano, which can enable the hot polymerization of HCN. Formose reaction may occur in temporary ponds formed by rainfall or hydrothermal processes that adjacent to volcanic vents, under mineral catalysis and geothermal heating. Temporary runoff from the surface of the volcanic body washed down reaction products, which can prevent overreaction that could lead to ribose degradation. In this process, polyphosphates can be mixed into the water stream and ribose may also be phosphorylated. Small-grained minerals and clays in water channels may separate ribose from other materials and carried away by the current. When this current flushes into a pond or basin containing adenine, the substrates for ATP synthesis are assembled, which will be condensed during the drying process driven by geothermal or sunlight. ATP can be accumulated during wet-dry cycles. The half-life of ATP at room temperature solution ranges from years to decades depending on the pH and ions [81], which could be longer in freezing or dry conditions. Therefore, ATP may have existed relatively stable on primitive Earth, waiting for interacting with other molecules.

## 4. Discussion

Because of the fundamentality and multiplicity of its roles, ATP has long been a focus of studies in various fields of life sciences. The previous content showed the possible pathway to ATP on primitive Earth. However, whether ATP assumed its present functions from the moment it was synthesized is still debatable. As a complex system, modern organisms are constructed by thousands of molecules, from simple H_2_O to genome DNA, ATP also depends on other molecules to function, which should also be the case in prebiotic chemical evolution.

As a component of RNA and cofactor of protein, ATP needs to cooperate with other nucleotides and amino acids to form functional polymers. HCN and its derivative acrylonitrile have been supposed to be sources of pyrimidine nucleotides, amino acids, and lipid precursors [76]. The related reactions may be occurred in different places with suitable conditions and the products are gathered through the water system. The wet-dry cycle environment in which ATP is produced is also appropriate for the condensation of nucleotides and amino acids. If ATP is present when the peptide is formed, it may bind to the latter and help them fold. This effect may have picked out some of the earliest structured peptides [82,83]. As evidence, ATP binds some oldest protein structures and conserved sequence fragments and is the most prevalent cofactor in protein structure space [2]. in vitro selection experiments using ATP as the bait in random sequence peptides obtained items with a similar structure or sequence motif to modern ATP binding proteins [84,85]. The inheritance of these proteins remains a problem because how RNA coding proteins and self-replication still does not have a solution. Recent studies showing the preference for interactions between different oligonucleotides and amino acids [86], as well as the modified nucleotides which are easier to be ligated [87], have shed new light on these problems. Moreover, the homochirality of RNA and protein, which is a significant issue in the origin of life, may also be achieved during the replication of RNA and ribozyme catalyzed peptide synthesis [88,89,90].

Interestingly, the in vitro selected peptides have ATPase activity, which is connected to the role of ATP as an energy currency. In modern organisms, the energy from ATP phosphate bonds hydrolysis is generally channeled to certain energy-consuming processes through specific proteins with ATPase activity. In addition to the ATP binding domain, these proteins normally have other domains to specifically bind substrates of the reaction that need to be powered. These proteins not only promote energy release but more importantly also enables precise control of energy flow. In the early stage of chemical evolution, these processes are unlikely to involve protein enzymes. Without the controller, the energy sources may be more extensive than the specific use of ATP. That is, the role of ATP as energy currency may be established as its binding proteins became dominant. Other simpler compounds such as pyrophosphate and acetyl phosphate may be more widely used in the prebiotic reactions [91]. Similarly, other simpler phosphorylation reagents may also have been widely used before the advent of kinases.

The recently identified hydrotrope function of ATP depends on its concentration at the millimolar level. This function may only be available and significant in the membrane-enclosed vesicles. In modern life, the membrane is a necessary condition for the stable supply of large amounts of ATP. ATP synthase is driven by the proton gradient inside and outside the membrane. The complex structure of ATP synthase implies its later origination. Moreover, the main function of hydrotrope is inhibiting biomacromolecule aggregation. If not confined in vesicles, it should also be difficult for macromolecules to reach concentrations where they can aggregate.

In summary, based on previous findings on the synthesis of ATP components, we constructed a terrestrial volcanism-dependent ATP synthesis pathway. Some other reactions that may play an important role in the prebiotic synthesis of ATP were also briefly described. After appearing on Earth, ATP may have first served as the component of macromolecules and driven energy-consuming processes together with other high-energy molecules. As enzyme-catalyzed reactions took over the original abiotic ones, ATP became the main direct energy source. Perhaps no later than the emergence of the enzyme system, the membranes that encapsulate it also appeared. To avoid the harmful aggregation of macromolecules in the crowded primitive cell, ATP got a new role as a hydrotrope. Beyond the fundamental functions in the origin of life, in modern organisms, ATP plays a role in antiaging [92], and may have an application in precision medicine by inspiring the identification of prognostic biomarkers and treatments for breast cancer [93].

An area less covered above is the possible contribution of extraterrestrial materials in ATP synthesis. In addition to the reactive phosphorus, ribose [94], nucleobases [95], and other abundant organics [96] have also been identified in meteorites, which may be involved in the prebiotic synthesis of ATP. The possible mechanism for how these compounds were generated in space was also reported recently [97]. The volcanism-based ATP synthesis pathway proposed in this paper may also be achieved beyond Earth. Primitive Mars is supposed to be somehow similar to primitive Earth. Primitive Mars was once geologically active, and Olympus Mons, the highest mountain in the solar system, was also created by volcanism [98]. Volcanic lightning can produce HCN in the carbon dioxide-nitrogen Martian atmosphere [99]. Geological activity may also be a source of formaldehyde [100]. In addition, Martian phosphate minerals have higher dissolution rates and phosphate release rates than common Earth phosphate minerals, which is conducive to the occurrence of phosphorus-related reactions [101]. Based on these substances, Earth-like planets such as Mars may be able to generate ATP according to the pathway we proposed. On the contrary, on smaller celestial bodies that cannot maintain geological activity and atmosphere, other ways are needed to produce ATP.

## Figures and Tables

**Table 1:** The difficulties and solutions in ATP synthesis.

Difficulties	Solutions
Synthesis of adenine requires a high concentration of HCN, but it is too active and unstable to accumulate on primitive Earth.	Store HCN in less reactive salts in mineral-rich water. Convert HCN to polymer under freezing or hydrothermal environments. Formamide generated during HCN hydrolysis can be concentrated under a volcanic environment. HCN independent pathways.
Synthesis of ribose with formose reaction requires a relatively high concentration of formaldehyde, but it is easily converted to formate and methanol in solution.	The continuously generated formaldehyde by the volcano-facilitated photochemical and electrochemical reactions. The continuously generated formaldehyde by hydrothermal vents.
The product of the formose reaction is complex, while ribose is not a major one.	Borate, silicate, and phosphate minerals can enhance ribose production. Clays can select ribose from complex reaction products. The glyoxylate scenario.
Ribose is unstable.	Preserve ribose by solid phase or low temperature. Using ribose as soon as possible.
The efficiency of condensing ribose and nucleobase is low.	Phosphorylated ribose is a better substrate. Construct nucleobase on phosphorylated ribose.
The phosphorylation of adenosine is inefficient in solution.	Trimetaphosphate could be a more effective phosphorylation reagent. Wet-dry cycles and metal ions promote the phosphorylation reaction.

## Data Availability

Not applicable.
